# Scaling the Superblock model to city level in Barcelona? Learning from recent policy impact evaluations

**Published:** 2022-12-31

**Authors:** Jaime Benavides, Sabah Usmani, Marianthi-Anna Kioumourtzoglou

**Affiliations:** Deptartment of Environmental Health Sciences, Columbia University Mailman School of Public Health, NY

**Keywords:** superblocks, health impact, urban environment, policy evaluation

## Abstract

Evaluating the intended and unintended environmental health impacts of urban policies is critical for designing and implementing policies that promote healthier urban living. This paper evaluates the Barcelona Superblock model — a policy aiming to reduce road traffic and increase other uses of the public space such as active mobility — during its design and recent implementation stages, with a focus on its traffic reduction benefits. Its design assumed that Superblocks would cover the entire city, potentially reducing overall road traffic by more than 20% due to the “evaporation” of road traffic from this large-scale reallocation of streets. This reduction is necessary to improve environmental health conditions in the city, where traffic density accounts for about 5,500 vehicles per km2 in the most congested areas. Recent estimations in the context of the COVID-19 lockdown found that a 25-30% reduction of road traffic is required to comply with European annual NO2 concentration standards. Thus, according to the impact evaluation of its design, an entire-city deployment of Superblocks would accomplish most of this necessary reduction. However, to date only three pilot intervention areas have been implemented in the city and two evaluations of their actual impacts provided complementary results: Superblocks are beneficial in pedestrianized streets but may be detrimental in streets influenced by the rebound effect of traffic redistribution. Previous studies have suggested that similar interventions reallocating road space from motorized vehicles to other uses can lead to traffic redistribution to nearby streets while reducing overall traffic levels on the whole network. Here, we argue that in cities with very high circulating traffic density, like Barcelona, Superblocks need to be accompanied by urban policies that reduce traffic in the city as a whole. Specifically, developing policies that could reduce overall traffic in the entire city — e.g., a congestion charge — could ease the implementation of Superblocks by freeing space from cars and providing economic resources to implement the necessary transformation of public space.

## Introduction

Many cities in Europe are currently implementing new urban models and policies that promote a shift from private cars to active transportation and green spaces (Nieuwenhuijsen, 2021). This trend has accelerated during the COVID pandemic (Combs and Pardo, 2021). The impacts of this drive towards healthier cities need to be assessed across multiple stages of the policy process, from inception to execution. This is vital for achieving real progress toward healthier and more equitable cities. The results of these evaluations can be used to raise awareness about the policy’s potential, and actual efficacy, and to inform the decision-making process to modify the design, if and when necessary, in order to substantially improve urban health. The environmental health impacts of a policy can be direct—when policy and impact take place on the same urban environmental feature (e.g., a congestion pricing scheme that charges a fee to drivers of motor vehicles in an urban area acts on traffic to reduce congestion)—or indirect—when the policy acts on an environmental feature but impacts a different one (e.g., a pedestrianization scheme impacting air pollution). Additionally, impacts can be intended (e.g., low emission zone impacts on reducing air pollution) or unintended (e.g., traffic redistribution due to extending green spaces in the city).

**Fig 1:**
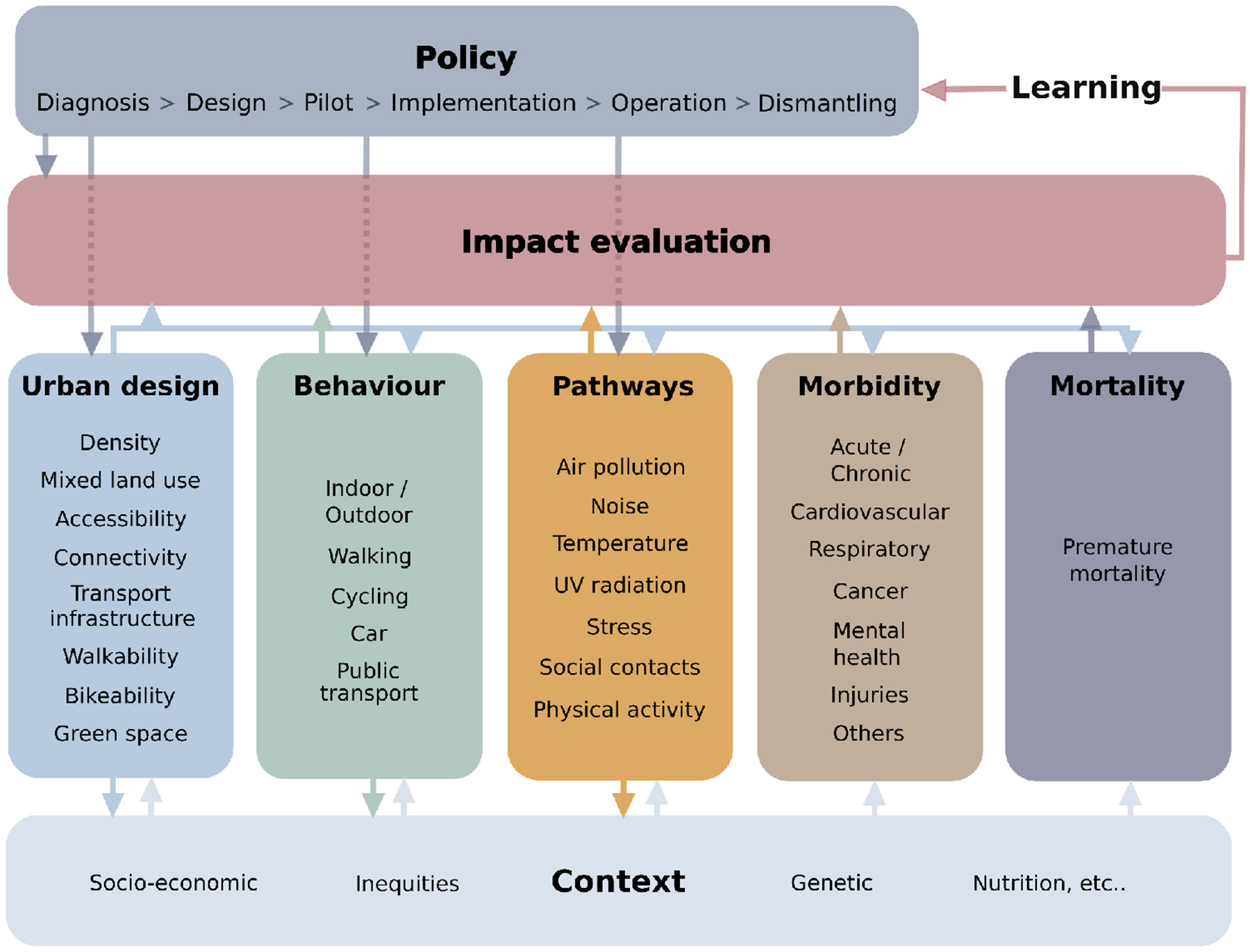
Impact evaluation of policies on urban environment and health at different stages of the policy process. Source: reproduced from Benavides et al. (2022)

Evaluations of policies can use quantitative or qualitative information in their impacts assessment. In general, quantitative policy impact evaluation involves estimating the impact of a policy on one or more variables, ranging from urban design features to public health (Fig. 1), e.g., the difference in noise exposure(s) under a policy versus a reality without policy. However, for studies examining already existing policies, both scenarios cannot co-occur, and for studies assessing the impact of unimplemented policies, both the policies and their impacts are not observable. In recent years, analysts have developed and used methods to examine these potential impacts using either simulation scenarios or observational evidence of policies that were at least partially implemented; these methods have been used separately (Benmarhnia et al., 2019; Baghestani et al., 2020) or combined (Nethery et al., 2020).

Since the end of the 20th century, several cities have implemented localized changes in their use of public space to shift focus from car-oriented towards pedestrian-friendly streets through a range of policies including pedestrian zones/streets, car-free days, parking restrictions, and other traffic-calming and control measures (Yasin, 2019; Yoshimura, 2022). In the 1960s, leading urban planning thinkers like Jane Jacobs questioned the dominance of road infrastructure and traffic and their impact on citizens’ daily life (Jacobs, 1968). In turn, the popularity of policies to pedestrianize city centers and high-traffic areas has been steadily growing. This is often coupled with investment in public transport, bicycle infrastructure, and improved accessibility/walkability to city services necessary to fulfill citizens’ mobility needs (e.g., school, healthcare). Contemporary urban models, like the 15-minute city, in which all main activities are reachable within a 15-minute walk or bicycle ride from the home, are a testament to this growing trend prioritizing non-motorized transport and mass transit (Abdelfattah, 2022). Yet most public space in a city like Barcelona (about 60%) continues to be used for road infrastructure dedicated to cars, including for traffic and parking. In recent years, several new urban models such as the Barcelona Superblock model are being implemented in different cities aiming to reconfigure the use of public space to reduce the use of motorized vehicles and increase public and active transport (e.g., walking and cycling), reduce air pollution, noise and heat island effects, and increase physical activity and social contact (Nieuwenhuijsen, 2021).

The Superblock model is a transformation of the road network which consists of interiors where motor vehicles and parking are restricted and priority is given to active mobility in public spaces. These spaces also contain recreational areas, meeting places, and extended green spaces. This paper analyzes recent policy evaluations of the Superblock model in Barcelona, Spain, with a focus on its effects on traffic reduction and redistribution, to gain insights on how to better scale this policy over the entire city.

## The case of Barcelona city

Barcelona acts as a hub of a network of interrelated cities in a densely populated metropolitan area (Eurostat, 2021). The city supports a high proportion of active transportation with its dry climate, compact and dense urban area, along with sustainable transport policies (BCC, 2014). Active transport such as walking and cycling accounted for 55% of the travel within the city in 2021, while public transport represented 24% of the overall trips (EMEF, 2021). Nonetheless, the Barcelona metropolitan area remains very dependent on passenger cars, decisively contributing to the high density of vehicles circulating in the city. Traffic density accounts for about 5,500 vehicles per km^2^ in the more congested areas, with the majority of these cars using diesel (Benavides et al., 2019). As a result of the intense use of public space by road traffic, levels of both noise and air pollution are high in the city (Mueller et al., 2017). For example, nitrogen dioxide (NO_2_) concentrations exceeded the European annual average limit value in the city of Barcelona from 2000 to 2019 (ASPB, 2021). NO_2_ is a combustion product primarily emitted by motor vehicles in urban environments, and is one of the pollutants linked to detrimental health effects in the city (ASPB, 2018). In a recent study by the local public health agency, these NO_2_ concentration levels were associated with about 929 premature deaths in Barcelona city in 2017 (ASPB, 2018).

Current urban and transport planning practices in Barcelona have substantial negative environmental and health impacts (Mueller et al., 2017). Urban policies are needed to reduce these negative health impacts and, in turn, several different traffic restriction policies are currently being applied in Barcelona: the Low Emission Zone (LEZ); partial pedestrianization of major urban corridors of the city after the COVID-19 pandemic, and the expansion of the Superblocks. The LEZ is applied in the entire city of Barcelona, inside “rondas” (i.e. the highways surrounding the city), and restricts entry to the oldest cars and motorbikes in the designated zone. The Barcelona Superblock model is an innovative urban model that has the potential to reduce negative impacts on urban health through the conversion of road infrastructure for traffic into accessways for public and active transport, in turn, increasing green spaces and physical activity, reducing air pollution, noise, and heat island effects (Rueda, 2019).

## The Barcelona Superblock model

**Fig. 2:**
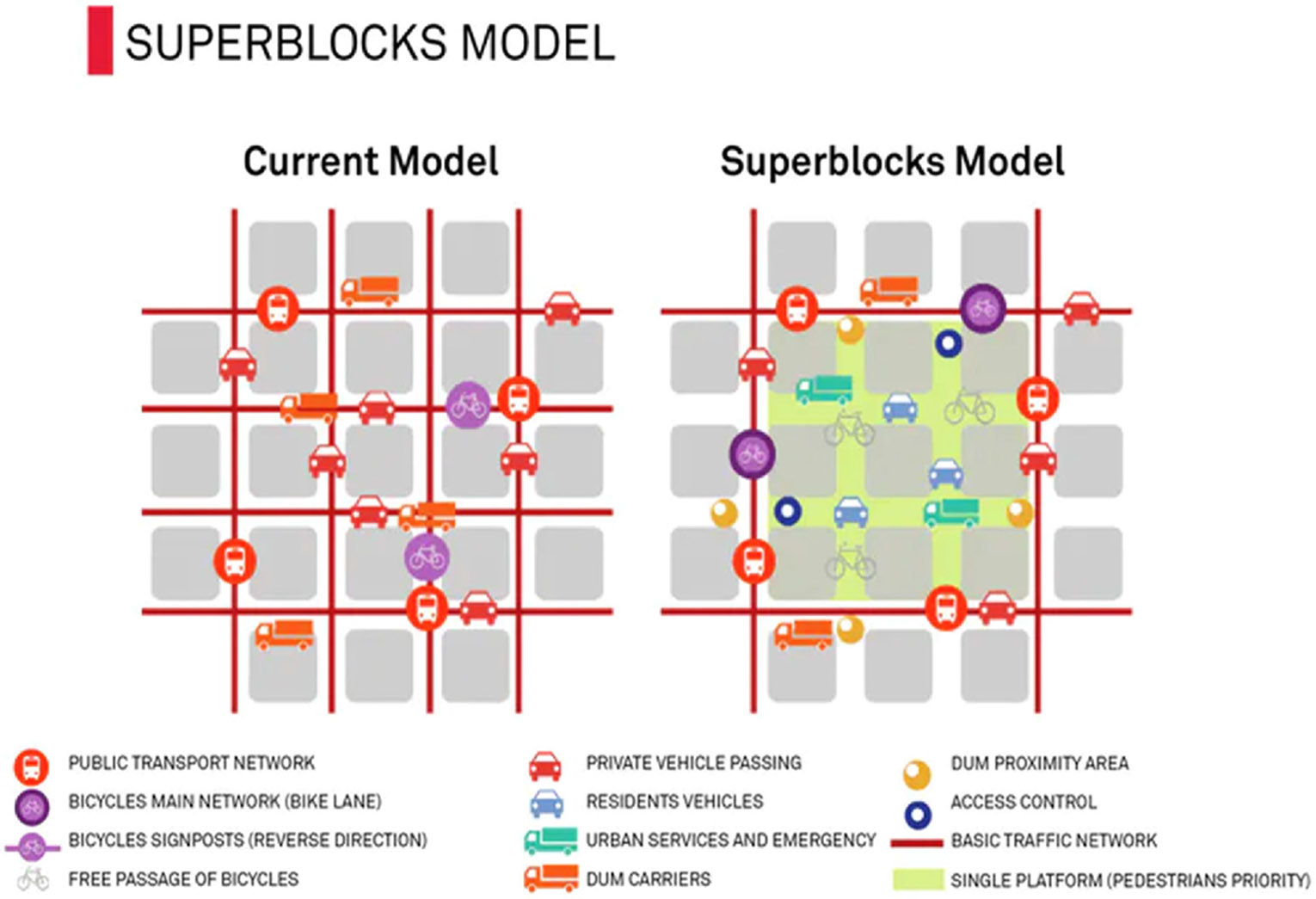
Difference between the prevalent road traffic and the original Superblocks model. Source: Reproduced from Barcelona (2014)

The Barcelona Superblock (Fig. 2) is an urban typology of modular street planning and design which consists of a network of roads with reduced access to motorized vehicles and parking within the zone designated as a Superblock. Historically Superblocks consist of nine city blocks (Fig. 2), with roads that prioritize pedestrian and non-motorized vehicle flow, combined with recreational areas, meeting places and targeted greenery enhancement. If applied at scale and throughout the city, the size of the Superblock model has the potential to accommodate the functional and morphological characteristics of the city and transform the maximal allowable public space away from road traffic while ensuring the functionality and organization of the system (Rueda, 2019). Several Superblocks have been implemented since 2016 in three of Barcelona’s neighborhoods: Poblenou, Sant Antoni, and Horta. The initial implementation in the Poblenou neighborhood found opposition by residents to this development in their local area (i.e., “not in my backyard” effect), with specific concerns in relation to potential risk of gentrification (López et al., 2020), but subsequent implementation of the model in the Sant Antoni and Horta neighborhoods was better received due to an increased involvement of local residents in the design and planning process (ISGlobal, 2022). Currently, a larger expansion of the Superblocks in Barcelona is being implemented in the form of ‘green axes’ with additional public squares at key intersections that prioritize users on foot and bicycles (Barcelona City Council, 2021). These axes would be located in the central district of Eixample, including a total of 33 km of streets and 21 new squares, providing a total of 3.9 hectares of new public spaces. If this project is fully deployed, in total, the Eixample district is expected to gain a total of 33.4 hectares of new pedestrian areas and 6.6 hectares of urban green areas (BCC, 2022). The current term of municipal government office is committed to develop 4 streets and 4 squares of this new program by May 2023 (BCC, 2022). Internationally, the Barcelona Superblocks model enjoys an increasing, generally favorable, international reputation, with press publications in many well-known international media platforms (e.g., The New York Times (2016), The Guardian (2019), Vox (2019)) and is being planned or implemented in other cities around the world. A recent scientific article by Eggimann (2022) examined the potential to implement the Superblock model in other cities with traditional grids as well as more heterogeneous urban layouts. The algorithm searched for streets that could be transformed to multifunctional streets following a geospatial approach. The author concluded that the Superblocks model policy can be transferred to other cities with sufficiently high population densities, finding that for some urban areas over 40% of the street network is potentially suitable for transforming road traffic space to Superblocks (Eggimann, 2022).

## Learning from the Superblock model impact evaluations

Policy evaluations can be conducted at multiple stages, following the policy process: diagnosis, design, pilot, implementation, operation, and dismantling (Benavides, 2022). Superblocks in Barcelona have been evaluated during the design phase and in the currently ongoing implementation phase. Here we summarize these recent evaluations and provide insights from this multi-stage perspective of the policy life cycle.

## Design stage

### Barcelona City Mobility Plan (2014).

The mobility and environmental impacts of the full city deployment of the Superblock model in Barcelona were evaluated in the Barcelona City Council Mobility Plan (2014). To simulate the reality without the policy (i.e., the base case scenario), and the scenario containing city-wide application of Superblocks, the authors of the study used a macroscopic traffic modeling system (TRANSCAD). The results revealed that the transformation of streets through the Superblocks model would lead to a 21% drop in circulating passenger cars from 2011 to 2018, decreasing congestion, and ensuring the levels of NO_2_ concentrations in all the monitoring stations remain below the EU annual air quality limit (i.e., 40 μg/m^3^). In recent years, the natural experiment of the COVID-19 lockdown allowed researchers to demonstrate that a reduction in traffic on working days of about 25 to 30% is required to avoid exceeding the EU annual NO_2_ limit in Barcelona (Querol et al., 2021). The 21% reduction in circulating passenger cars would primarily come from the reduction in public space used for road traffic, which would be drastically reduced from 1483,6 ha to 815 ha (−45%) (Rueda, 2019). The main limitation of this modeling exercise is that it followed a macroscopic approach, which provides overall results at the city level but it does not inform about the most affected streets or about possible rebound effects. Additionally, simulated scenarios did not include incremental partial implementations of the policy and their potential consequences for the traffic flow.

### Mueller et al. (2020).

This study evaluated the health impacts from the Superblock model applied to the entire city of Barcelona (i.e., 503 Superblocks). The authors used the UTHOPIA model of Health Impact Assessment (Mueller et al., 2017) to estimate the health impacts of the Superblocks model using metrics previously simulated in the Barcelona City Mobility Plan. The authors assessed the impact of the policy using five main environmental pathways: road traffic noise, green spaces, air pollution, urban heat island and transport-related physical activity (Mueller et al., 2020). For each environmental pathway (i.e., air pollution (NO_2_), road traffic noise, green space, transport-related physical activity, and reduction of the urban heat island effect), they compared a base case scenario (i.e., without Superblocks) representing the year 2016 and another scenario including Superblocks all over the city. They assumed a 19.2% reduction in circulating passenger cars based on traffic simulations made for the Barcelona Mobility Plan (2014) and estimated an overall reduction of 24% in the annual mean NO_2_ concentrations. In the subsequent health impact analysis, these NO_2_ reductions contributed the greatest proportion of preventable premature deaths among the examined exposures as a result of the full-scale Superblocks policy implementation (i.e., 291 out of 667 preventable premature deaths due to Superblocks). This study concluded that Superblocks would greatly increase green spaces and physical activity, while reducing air and noise pollution. The 667 preventable premature deaths and the average increase in life expectancy for the adult population of almost 200 days per year would amount to a great saving in health costs, estimated at EUR 1.7 billion per year. This study followed the same approach for mobility simulations as the one used in the Barcelona City Council Mobility Plan (2014), and thus, it is also limited by its use of an approach that does not provide the spatially resolved detail of expected traffic changes and does not include analysis of the phased/partial implementation of the full-scale, city-wide policy.

## Implementation stage

### Superblocks evaluation by the local agency for public health (ASPB, 2021).

**Fig. 3:**
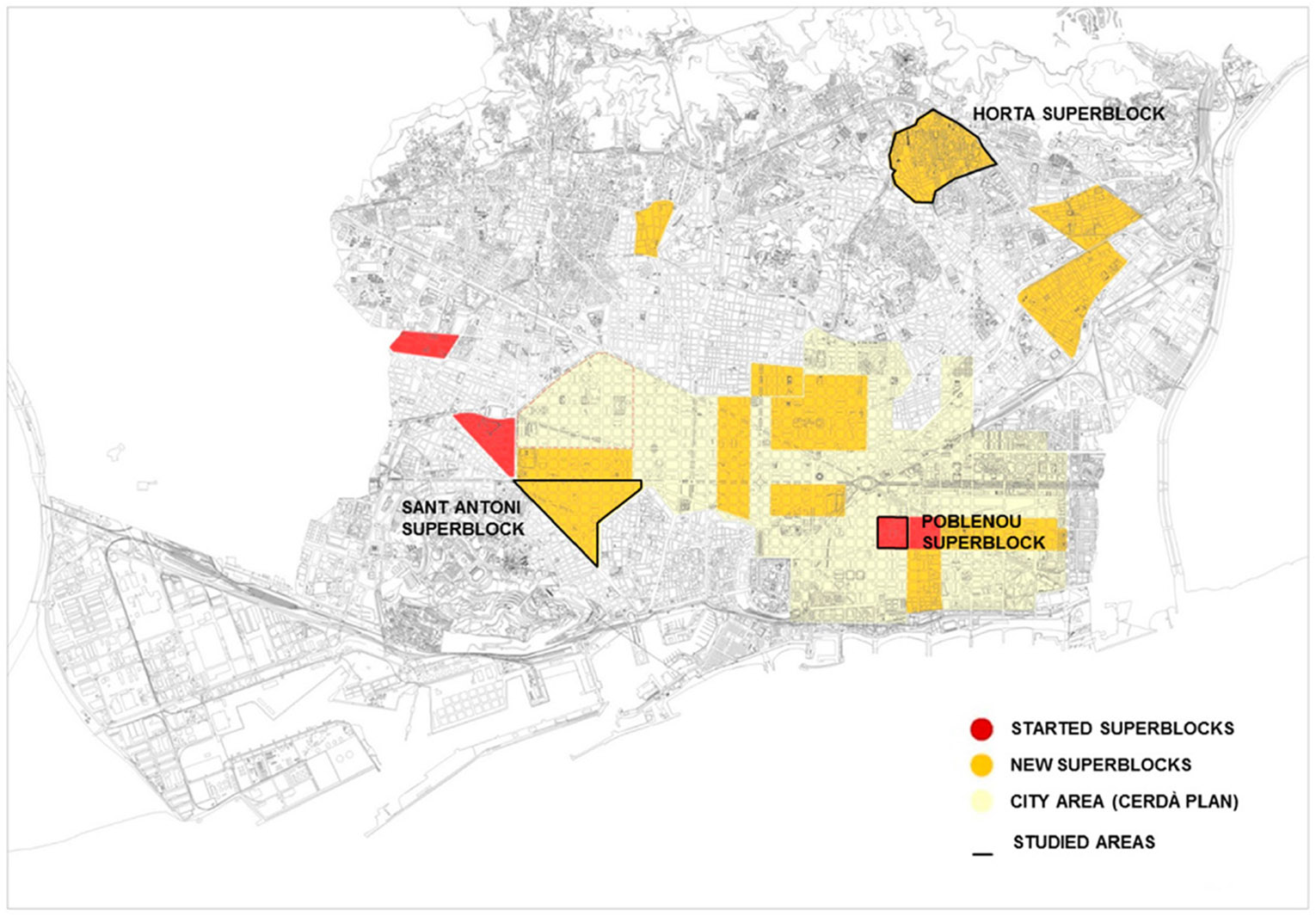
Barcelona Superblocks, 2017–2019. Source: reproduced from Palència et al. (2020)

This study assessed the potential environmental and health impacts of the Superblocks implemented since 2016 in three Barcelona’s neighborhoods (Poblenou, Sant Antoni. and Horta; Fig. 3) (ASPB, 2021). They used both quantitative and qualitative techniques and the complete methodology is described in Palencia et al. (2020). Among other tools, they used outdoor air quality measurements, an observational study of physical activity, a health survey conducted both pre and post intervention, and a qualitative study with focus groups.

Most benefits in this study are reported in qualitative terms: residents from pedestrianized streets reported better rest, increased socialization, and less perceived noise and air pollution (ISGlobal, 2022). Quantitatively, the change in air pollution varied by intervention area according to this study. In Sant Antoni, measurements gathered at pedestrianized streets showed a 25% reduction in NO_2_ levels and a 17% reduction in PM_10_ levels. In Horta, no significant changes in pollutant levels were measured (ASPB, 2021). In this evaluation, no road traffic, air pollution, or noise measurements were gathered outside the intervention area (e.g., in parallel streets where traffic could be redistributed). Rodríguez-Rey et al. (2022). This study used the Barcelona Virtual Mobility Lab (VML) model (Montero et al., 2018) to simulate scenarios of different urban policies coupled with a multiscale (i.e., from regional to street level) air quality model (CALIOPE-Urban, Benavides et al., 2019). The VML uses a VISUM traffic simulator (PTV Group, 2019). This technique provides information of the change of traffic parameters, such as number of circulating vehicles and speeds per road segment given different scenarios. The authors assessed the traffic, emissions and air quality impacts of the Superblocks implemented since 2016, the Low Emission Zone (LEZ), and other pedestrianization interventions derived by the COVID-19 pandemic in Barcelona. This modeling system allows the estimation of policy impacts on traffic, emissions, and air pollution at high spatial resolution (20 m × 20 m). In the study, both individual and collective reductions from the mentioned policies were assessed, finding that these impacts are still insufficient to meet the EU annual mean NO_2_ concentration standards. For example, the LEZ would reduce NO_X_ emissions by around 13% and a complementary reduction of 25% in traffic demand throughout the city would be required to reduce emissions by 30%. Specifically evaluating Superblocks, they found that this intervention produced only localized street-level NO_X_ changes, both positive and negative. NO_X_ emissions in affected streets (i.e., both pedestrianized and adjacent streets) varied between −17% and +17%. In detail, emissions in one of the urban corridors with higher traffic load and emissions (i.e., Aragó Street) showed an average reduction of −17% in NO_X_ emissions with a traffic flow reduction of −24%. On the other hand, increases up to +17% in NO_X_ emissions were estimated in other adjacent streets (e.g., Tarragona or Viladomat Streets) as a consequence of traffic redistribution, which resulted in an increment of traffic flow of +30% and +125%, respectively (Rodríguez-Rey et al., 2022). This study provides a more holistic view of the impact of the implemented Superblocks in Barcelona by investigating both intended and unintended impacts. ISGlobal (2022) highlights an important limitation of this study which is that it does not assume an overall reduction of road traffic as a consequence of change in behavior by car drivers disincentivized by the reduction in space for road traffic, which they claim is the most likely scenario.

## Evaluation insights

The recent impact evaluations analyzed in this work show a great potential to improve environmental health in Barcelona through the implementation of the 503 Superblocks intended to fundamentally transform the urban fabric of the entire city (Rueda, 2019). However, current studies assessing the environmental and health impacts of implemented Superblocks show limited and complementary impacts: Superblocks may be beneficial in streets where road traffic has been limited but their influence may be detrimental in nearby streets where traffic is likely to be redistributed. In addition, the current pace of transformation (3 Superblocks in more than 5 years) would take several decades to be operational at city scale and to bring to reality the projected environmental health benefits estimated by Mueller et al., (2020). Also, given these recent evaluations, there is a clear disconnect between the impacts of these two stages of the Superblocks model (i.e., design and current implementation), neglecting the continuity of the policy life cycle. For instance, the studies do not address the challenges and opportunities of implementing the Superblock typology across the entire city and the best practices on how to do this effectively. A more realistic approach would take into account a phased implementation of the Superblocks which would necessarily require periods of partial application of the policy, leading to traffic rerouting scenarios not captured in the design stage’ studies. Assuming that the full city deployment of the Superblock model is attainable and given its potential benefits, a faster and city-scale implementation of the original Superblock model (i.e., 503 Superblocks) is urgently needed (ISGlobal, 2022). However, additional aspects may need to be considered to foster effective implementation.

## The need for city-wide traffic reductions

**Fig. 4:**
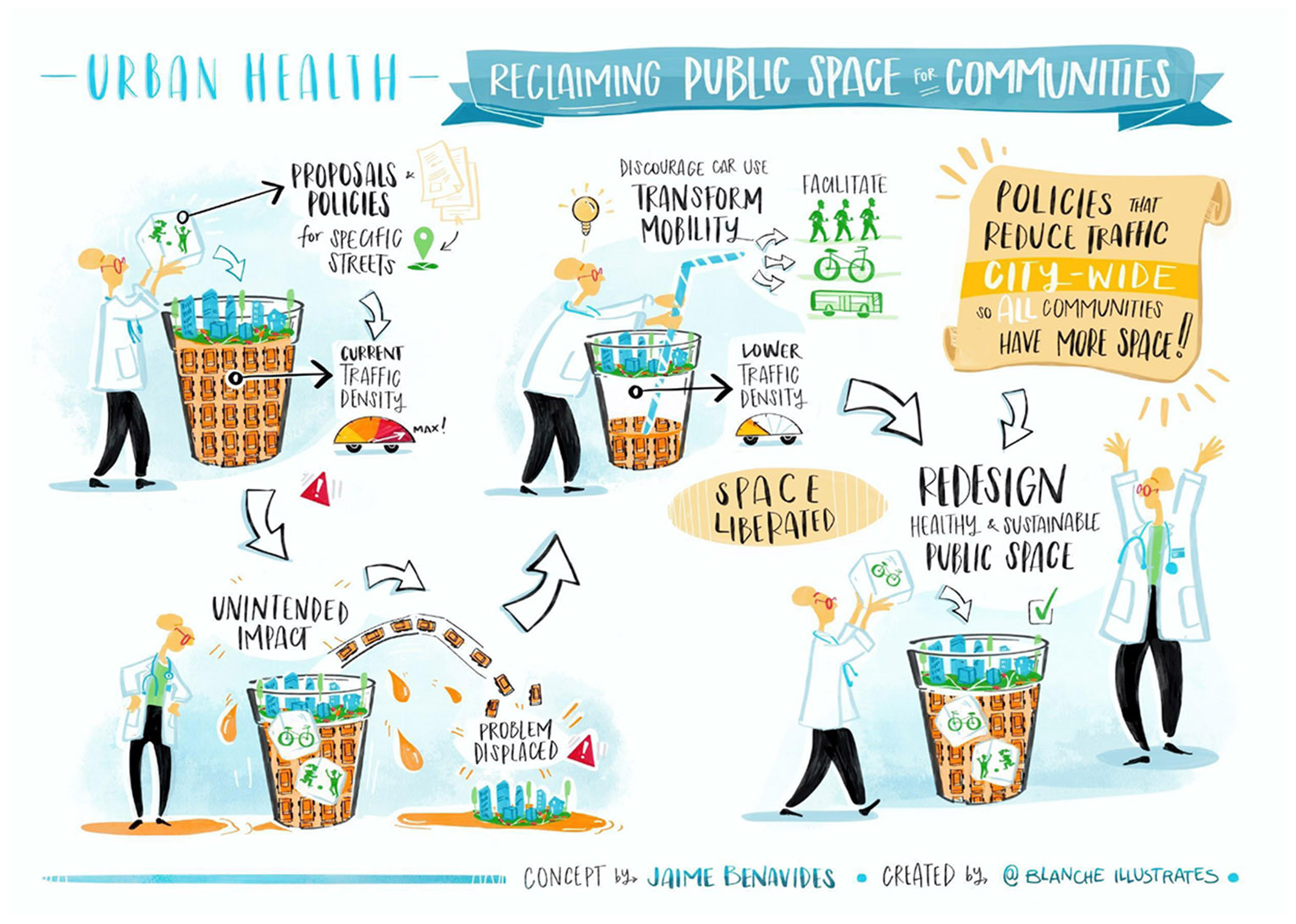
Policies that reduce traffic city-wide so all communities have more open space. Source: Author and Blanche Ellis (illustrator).

A recent study in the context of COVID-19 lockdown estimated that a traffic reduction of 25-30% is necessary to respect european air quality standards in Barcelona (Querol et al., 2021). Also, the initial Superblock model design would require a traffic reduction of about 20% to be functional (BCC, 2014) and effective in improving environmental health conditions (Mueller et al., 2020). Previous studies suggested that similar interventions reallocating roadspace from motorized vehicles to pedestrians and other non-motorized uses, also, incur in traffic redistribution to nearby streets but reduce overall traffic levels on the whole network (Cairns et al., 2002). This traffic evaporation may not be sufficient to drastically reduce the overall road traffic in a city with very high vehicle density like Barcelona. In addition, the slow deployment of the Superblock model may perpetuate rebound effects due to redistribution of traffic, undermining its potential to improve environmental health conditions in the city in the short or mid term. As a consequence, reducing traffic density at city scale in Barcelona is necessary to improve citizens’ health, in general, bolstering the case for the full-deployment of the Superblock model (Fig. 4). Thus, combining Superblocks with other policies that have already achieved such traffic reductions at the city level in other urban areas could ease the path towards a healthier city in Barcelona and in other cities with similarly high road traffic volumes that are detrimental for human health.

Superblocks can be implemented in conjunction with other policy interventions to reduce urban traffic. A recent scientific review by Kuss et al. (2022), assessed a number of such strategies and concluded that congestion charge was one of the most effective policies for achieving profound reductions in overall traffic and a modal shift to more sustainable and healthy modes (Kuss et al., 2022). Congestion charge is a road transportation policy which aims to limit traffic congestion during peak periods, in turn diverting or reducing vehicle traffic in select streets or geographically defined zones in urban areas through a fee or toll for vehicles entering the area. For example, in Stockholm the congestion charge introduced in 2007 achieved an overall traffic reduction of 22% in the first year of implementation in an area comparable to the current Barcelona LEZ (Eliasson, 2014). In London, the congestion charge led to a decrease of 33% in overall traffic following introduction of the charge.

This traffic reduction remained stable but it was followed by a rebound over time to previous congestion levels due to factors such as reduced road space for motor vehicles (Metz, 2018). In Spain, this policy has not yet been implemented and was not supported by legal instruments. However, the recent draft of the future Sustainable Mobility Law contains a legal authorization to allow the municipalities willing to introduce a congestion charge in the already delimited low emission zones (Spanish Government, 2022). In addition, citizen groups/non-profit organizations in Barcelona focused on clean air and safe active transportation have developed a proposal to advocate for a congestion charge in the city (PQA, 2022). In their proposal, car drivers would be required to pay €4 to go through the current Barcelona LEZ during daytime hours on weekdays, and the net revenue generated will mainly be used for investment in public transport and public health systems (PQA, 2022).

Kuss et. al.’s (2022) review suggests an advantage to deploying multiple traffic restriction interventions and policy instruments to reduce traffic (Kuss et al., 2022). In Barcelona, there is an opportunity to use a multi-policy approach that takes advantage of the Superblocks and the congestion pricing program to reduce city-wide traffic density, effectively reassigning roads from vehicular traffic to pedestrians and active transport modalities. The two approaches are inherently complementary. Traffic restricting policies, such as congestion charge, aim to reduce road traffic through pricing mechanisms. Unlike policies that restrict vehicle access directly on specific streets (e.g., Superblocks), congestion charge aims to reduce congestion by reducing the overall number of vehicles in the areas where it is implemented. The two interventions in conjunction could reinforce each other and contribute to easing the transition between space for road traffic and other uses at city level. In addition, the London experience could be helpful to foresee potential limitations of combining the Superblock model with a congestion charge policy because traffic congestion could rebound over time, even with a sustained reduction of circulating vehicles, due to a decrease of space allocated for motor vehicles. Lastly, congestion pricing could also provide economic resources for the necessary investment in transforming the use of public space in the Superblock model, fostering public and active transportation.

## Conclusions

The aim of this study is to review the recent policy evaluations assessing the impact of the Superblock model using the case of Barcelona, with a focus on the impacts on road traffic, and to discuss challenges to make it scalable to the entire city based on these evaluations. When evaluating the design of the Superblock model at the city scale, prior studies reported that this policy has a great potential to improve environmental health conditions in the city. However, these studies assumed a traffic evaporation all over the city of more than 20%, which may be too optimistic. Evaluations assessing the impacts of the recent implementation of Superblocks in three intervention areas in Barcelona found that Superblocks are beneficial locally where road traffic is drastically reduced, but may divert traffic and create subsequent issues in streets affected by rebound effects. Future evaluations of this policy should include the estimation of unintended impacts by reinforcing the evaluation methodology with traffic, noise and air pollution measurements in streets where traffic may be redistributed. In addition, a combination of the Superblock model with a policy aiming to reduce overall road traffic in the network, such as the congestion charge, may facilitate the implementation of Superblocks and provide economic resources towards the necessary transformation of the public space.

## Figures and Tables

**Table 1:** Summary of approaches to evaluate the impact of Superblocks in Barcelona at different stages of the policy process. Estimated environmental and health impacts shown in italics.

Policy stage (Scale)	Category	Method(s)	Urban design/ behavior	Pathways	Health	Citations
Design (Full city)	Simulation	Transportation model, dispersion model	Road traffic (*−21%*); public space for road traffic (*−45%*)	Air pollution (*NO*_*2*_ *conc. < EU annual limit*)	-	Barcelona City Mobility Plan (2014)
		Transportation model, surveys, dispersion model, noise propagation model, local temperature model, exposure-response relationships (ERRs)	Road traffic (*−19.2%*); green space in Eixample neighborhood (*+201%*)	Air pollution (*−24% in annual NO*_*2*_ *conc*.); road traffic noise (*−5.4% in annual mean*); temperature (*−1°C*); bicycle trips (*+20%*), walking trips (*+9%*)	*667 preventable premature deaths (44% due to NO*_*2*_ *, 24% to road traffic noise; 18% to heat, 9% to green space, 5% to physical activity)*	Mueller et al. (2020)
Implementation (localized)	Observational	Pre-post air quality and noise measurements, surveys, qualitative study with focus groups	-	Air pollution and noise (e.g., *Sant Antoni −25% in NO*_*2*_ *levels, −17% in PM*_*10*_ *levels, −5% daytime noise, no change in night noise)*	-	Local agency for public health (ASPB, 2021)
	Simulation	Transportation model, dispersion model	-	NO_X_ emissions positive and negative changes (±17%)	-	Rodríguez-Rey et al. (2022)
